# Minimally invasive treatment of patients with bronchobiliary fistula: a case series

**DOI:** 10.1186/1752-1947-3-23

**Published:** 2009-01-23

**Authors:** Unal Aydin, Pinar Yazici, Fatih Tekin, Omer Ozutemiz, Ahmet Coker

**Affiliations:** 1 Department of General Surgery, Ege University School of Medicine, Bornova, 35100, Izmir, Turkey; 2 Division of Gastroenterology, Ege University School of Medicine, Izmir, Turkey

## Abstract

**Introduction:**

Bronchobiliary fistula is an uncommon complication secondary to hepatobiliary surgery. Bilioptysis is a pathognomic finding for bronchobiliary fistulas. Diagnosis may be easily established in the light of clinical history, which can be aided by imaging studies to pinpoint the exact location. Some diagnostic procedures such as endoscopic retrograde cholangiopancreatectomy are also useful for treatment.

**Case presentation:**

We present three Turkish patients with bronchobiliary fistula secondary to previous hepatic surgery due to hydatid cyst in two, a 19-year-old and a 47-year-old man, and iatrogenic trauma of the common bile duct by endoscopy in a 35-year-old man. All of the patients were successfully treated by minimally invasive methods including percutaneous drainage and endoscopic retrograde cholangiopancreatography.

**Conclusion:**

We suggest that bronchobiliary fistula could be managed through conservative treatment methods which do not require in-hospital follow-up, particularly in uncomplicated cases. Otherwise, surgical management can be unavoidable.

## Introduction

Bronchobiliary fistula (BBF) is a relatively unusual entity, which is defined as an abnormal communication of the biliary system with the bronchial tree resulting in bilioptysis (bile-stained sputum). It was first described by Peacock in 1850 [1]. Patients with BBF usually present with expectoration of bile as a cardinal symptom. Therefore, the diagnosis is based on clinical symptoms as well as clinical history. The underlying factors are hepatic trauma, previous hepatobiliary surgery, hydatid disease, and other hepatic disorders [2,3]. There are still no definite guidelines for the optimal management of this rare condition because most of the reports on BBF are only in the form of case reports. In a 5-year period, we encountered two cases with BBF secondary to hepatobiliary surgery and one due to previous endoscopic intervention. All of the patients were of Turkish ethnic origin.

## Case presentations

### Case 1

A 19-year-old boy was admitted to the hospital with symptoms of abdominal tenderness located in the epigastric region and vomiting. Ultrasonography (US) demonstrated a hydatid cyst nearly 12 cm in size. The patient underwent cystotomy through a drainage procedure. His recovery was uneventful and he was discharged from the hospital on the fifth postoperative day. One month later, the patient presented with cough productive of greenish sputum. The cough had persisted for 2 weeks without any colored sputum. The temperature of the patient was 38.1°C. No dyspnea or jaundice was detected, but there was mild tenderness in the right quadrant. Laboratory studies revealed increased levels of alkaline phosphatase (ALP), bilirubin, and white cell count. Bronchoscopy was performed demonstrating bile in the bronchial tree, and US revealing density in the right lower lung and fluid collection in the subdiaphragmatic area. Therefore the fluid collection was drained percutaneously, and then antibiotherapy was initiated. In the early days of drainage, approximately 500 cc bile flow per day was observed. Because of persistence of the symptoms 4 days later, an endoscopic retrograde cholangiopancreatography (ERCP) was performed, and a biliary stent was placed after sphincterotomy. Bile drainage had almost stopped flowing 10 days later. The control US revealed no more fluid collection, and therefore the drainage catheter was removed after 2 weeks. The patient did well and had no recurrence of bilioptysis in the follow-up period of 15 months.

### Case 2

**Figure 1:**
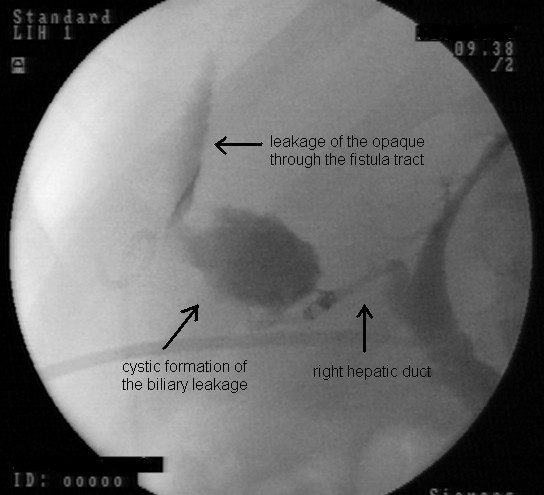
**Fistula tract between the biliary tree and fluid collection resulting from bile leakage through the right hepatic duct**.

ERCP for choledocholithiasis, and sphincterotomy were performed on a 35-year-old man. Three weeks later, he presented to our hospital with abdominal distension and pain located particularly on the right side of the abdomen. The patient also suffered from productive cough. On US and computed tomography (CT) images, an 8 × 10 cm subdiaphragmatic fluid collection and a dense area in the right lower lung were observed. ERCP demonstrated a fistula between the biliary tree and the lower right lobe of the lung (Figure 1). A nasobiliary drainage catheter was inserted. In addition, management included a catheter for percutaneous abdominal drainage and antibiotherapy. Fluid collection disappeared 2 weeks after catheter insertion, and the catheter was removed 5 weeks later. The patient had an uneventful recovery and has had no recurrence of bilioptysis within an 11-month follow-up period.

### Case 3

Cholecystectomy and cystectomy due to hydatid cyst of the liver were performed on a 47-year-old man. One week later, he presented with right abdominal pain and fever. Abdominal CT conducted at another hospital demonstrated a 6 × 8 fluid collection in the right lobe of the liver. The patient was managed with percutaneous abdominal drainage and antibiotics. Over the next few months, the fluid collection decreased in volume, but there was infiltration to the lungs. Bilioptysis developed and the patient was referred to our institution. The laboratory findings revealed elevated cholestatic enzymes and mildly elevated liver function tests. ERCP demonstrated a fistula between the biliary tree and the lower right lobe of the lung. Radiological coil embolization of the fistula and insertion of an internal and external drainage catheter were performed. Ten days later, ERCP was repeated due to persistent cholangitis, and sphincterotomy with biliary stent insertion was performed. After 2 weeks of antibiotherapy administration, the patient was discharged from the hospital. One week later, the catheter was removed upon confirmation of resolution of the fluid. No recurrence occurred during 7 months of follow-up.

## Discussion

The presence of bile on XXXthoracentesis of a pleural effusion and bilioptysis are pathognomonic for BBF [1]. BBF may be caused by several hepatobiliary diseases, including hydatid disease, cholelithiasis and acute cholecystitis, chronic pancreatitis, emphysema of the pleura, liver metastases, congenital fistula and abdominal trauma surgery [4]. The mechanism of transdiaphragmatic extension of biliary fistula to develop into a BBF is still controversial. One reason could be an injury of the diaphragm during trauma or surgery [3]. However, in our patients, the long latency period between surgery and occurrence of the BBF suggested that a diaphragm injury was unlikely. Nevertheless, in the second patient, an injury to the common bile duct was the reason for the subdiaphragmatic fluid collection. One of the mechanisms is that biliary obstruction produces an inflammatory reaction in the subdiaphragmatic space and subsequent rupture into the bronchial system. However, another mechanism is described as damage caused by the hydatid cysts spreading through the diaphragm into the pleural cavity and thus resulting in a BBF [5].

Early diagnosis of BBF is essential in its management. A delayed diagnosis results in serious complications. However, persistent BBF causes severe lung damage by bile flow into the bronchial tree and even necrotizing pneumonia: such a frightening complication should be kept in mind in the management strategy of the disease. In most cases, imaging tests are required to confirm the diagnosis and to clarify the anatomic situation [4]. The most important imaging techniques for assessment of a bronchobiliary fistula are CT, which is the most commonly used imaging technique in evaluation of the upper abdomen as well as the chest, and magnetic resonance cholangiography [1,3,6,7]. Abdominal ultrasonography is the primary investigation technique in our management policy. Both CT and US are inefficient in determining the exact location of the BBF, which is very important in clarifying the surgical method. However, US is used to detect any biliary collections or abscesses and may be useful in treatment by percutaneous drainage. As in our series, these interventions may not be adequate to eliminate the main reason. Hepatoiminodiacetic acid (HIDA) scanning and percutaneous transhepatic cholangiography (PTC), particularly for patients with external percutaneous biliary fistulization, are included among other diagnostic tools [8,9]. ERCP is also thought to be useful in demonstrating the fistulous tract and identifying distal biliary obstruction, with more benefit involving the possibility of applying several therapeutic procedures such as stent insertion or dilatation of the biliary ducts. Furthermore, endoscopic sphincterotomy may be undertaken. In our series, ERCP and interventional radiography such as external and internal drainage of the bile ducts were considered to be adequate. In case of pneumonia, bronchoscopy should be performed in order to maintain a sputum sample to detect the causative factor and to determine the origin of the fistula.

There is still no optimal therapy for BBF. Consequently, every clinic determines its own procedure considering the experience and facilities available at the clinic. To determine the surgical procedure before the operation, ERCP is useful in both maintaining negative pressure to relieve overpressure in the biliary tract and performing nasobiliary drainage at the same time, enabling the control of the bile flow along an open fistula tract following the path of least resistance. In our three patients, ERCP including nasobiliary stent insertion and endoscopic sphincterotomy were performed concomitantly because of persistent symptoms. It has been recommended that endoscopic sphincterotomy may be practiced when the symptoms persist within 72 to 96 hours of tube thoracostomy due to advanced pleural effusion or percutaneous drainage of sepsis [10,11]. Fortunately, none of our patients needed a tube thoracostomy. Despite the effective nasobiliary drainage and PTC, cholangiography is useful in identifying bile leakage in the case of persistent bile flow by PTC. If there is no leakage, PTC should not be removed earlier than at least 1 month. Despite using all of the options, when medical treatment fails, BBF should be managed by endoscopic sphincterotomy before recourse to surgery [10]. Endoscopic sphincterotomy can ensure bile flow into the duodenum, eliminating any possible obstruction. Biliary decompression can also be obtained by temporary transampullary stenting. In our patients, after sphincterotomy with nasobiliary drainage, the fluid collection and external catheter volume decreased gradually. Thus, the pressure in the fistula tract, which inhibits the closing of the tract completely, was reduced. Before advances in minimally invasive surgery (endoscopic interventions), treatments for bronchobiliary fistula have traditionally been surgical. Surgical strategies include simple drainage of the subdiaphragmatic abscess, resection of the fistula tract and involved liver and lung tissue [6,12,13]. Surgical repair of the liver and diaphragm through a thoracotomy is preferred. In particular, complicated patients and those with benign etiology, recurrent BBF after conservative treatments or persistent fistula are candidates for surgical treatment. Thoracic surgery is recommended for complicated BBF in order to avoid serious pulmonary damage. Furthermore, a delayed surgical treatment unavoidably leads to a major lung resection [5]. This could be technically difficult and requires experience in thoracic surgery. For the BBF secondary to malignancy, a conservative approach is recommended [6]. However, no surgical procedure was required in our patients.

## Conclusion

We suggest that BBF could be managed through conservative treatment methods which could be considered as an initial feasible approach to BBF, because these noninvasive methods are regarded as efficient and generally do not necessitate in-hospital follow-up. In case of complications and/or failure of conservative techniques, the patient can be referred to a thoracic/abdominal surgery unit. A multidisciplinary management involving general surgery, interventional radiology, gastroenterology, and thoracic surgery may provide the desired outcome.

## Abbreviations

ALP: alkaline phosphatase; BBF: bronchobiliary fistula; CT: computed tomography; ERCP: endoscopic retrograde cholangiopancreatography; HIDA: hepatoiminodiacetic acid; PTC: percutaneous transhepatic cholangiography; US: ultrasonography.

## Consent

Written informed consent was obtained from the patient for publication of this case report and any accompanying images. A copy of the written consent is available for review by the Editor-in-Chief of this journal.

## Competing interests

The authors declare that they have no competing interests.

## Authors' contributions

UA made substantial contributions to the conception and design, acquisition of data and managed the cases.

PY made substantial contributions to the acquisition of data, was involved in drafting the manuscript and revised it critically for important intellectual content.

FT made substantial contributions to obtaining the data and helping to manage the cases.

OO gave final approval of the version to be published and made substantial contributions to the interpretation of data

AC made substantial contributions to the conception and design and drafting of the final version of the manuscript.
